# Model-based small area estimation methods and precise district-level HIV prevalence estimates in Uganda

**DOI:** 10.1371/journal.pone.0253375

**Published:** 2021-08-06

**Authors:** Joseph Ouma, Caroline Jeffery, Colletar Anna Awor, Allan Muruta, Joshua Musinguzi, Rhoda K. Wanyenze, Sam Biraro, Jonathan Levin, Joseph J. Valadez

**Affiliations:** 1 Division of Epidemiology and Biostatistics, School of Public Health, University of Witwatersrand, Johannesburg, South Africa; 2 METRe Group, Department of International Health, Liverpool School of Tropical Medicine, Liverpool, United Kingdom; 3 Data Science and Informatics Branch, Centers for Disease Control and Prevention, Uganda; 4 AIDS Control Program, Ministry of Health, Uganda; 5 Department of Disease Control and Environmental Health, Makerere University School of Public Health, Kampala, Uganda; 6 ICAP at Columbia University, Nakasero, Kampala, Uganda; University of Salamanca, SPAIN

## Abstract

**Background:**

Model-based small area estimation methods can help generate parameter estimates at the district level, where planned population survey sample sizes are not large enough to support direct estimates of HIV prevalence with adequate precision. We computed district-level HIV prevalence estimates and their 95% confidence intervals for districts in Uganda.

**Methods:**

Our analysis used direct survey and model-based estimation methods, including Fay-Herriot (area-level) and Battese-Harter-Fuller (unit-level) small area models. We used regression analysis to assess for consistency in estimating HIV prevalence. We use a ratio analysis of the mean square error and the coefficient of variation of the estimates to evaluate precision. The models were applied to Uganda Population-Based HIV Impact Assessment 2016/2017 data with auxiliary information from the 2016 Lot Quality Assurance Sampling survey and antenatal care data from district health information system datasets for unit-level and area-level models, respectively.

**Results:**

Estimates from the model-based and the direct survey methods were similar. However, direct survey estimates were unstable compared with the model-based estimates. Area-level model estimates were more stable than unit-level model estimates. The correlation between unit-level and direct survey estimates was (*β*_1_ = 0.66, *r*^2^ = 0.862), and correlation between area-level model and direct survey estimates was (*β*_1_ = 0.44, *r*^2^ = 0.698). The error associated with the estimates decreased by 37.5% and 33.1% for the unit-level and area-level models, respectively, compared to the direct survey estimates.

**Conclusions:**

Although the unit-level model estimates were less precise than the area-level model estimates, they were highly correlated with the direct survey estimates and had less standard error associated with estimates than the area-level model. Unit-level models provide more accurate and reliable data to support local decision-making when unit-level auxiliary information is available.

## Introduction

Model-based small area estimation (SAE) methods can help monitor the impact of public health interventions and appropriately allocate resources in small geographical areas where the domain-specific sample is not large enough to support direct estimates of adequate precision. Other terms used to refer to small geographical areas include “small domain” or “local area” [1]. SAE methods link a study/outcome variable with auxiliary data from other sources to produce more precise indicator estimates than direct-survey estimates (i.e., design-based estimates based on survey data alone) for the target local area.

Sources of auxiliary information may include routine administrative data from Health Information Systems (HMIS), censuses or other surveys. However, external covariate data are limited for predicting local area estimates. For example, routine data only capture information from individuals who interact with health facilities [2–4]. A study combining national population survey and routine data found a 28 improvement in the precision of the estimates [5]. General population censuses, on the other hand, are conducted decennially and do not capture information in the interim or information about HIV/AIDS risk factors such as number of sexual partners or condom use during last high-risk sex [6–12], rendering these censuses unsuitable for assessing outcomes that change rapidly. Annual HIV risk factor surveys with adequate level of precision, such as in the community Lot Quality Assurance Surveys (LQAS) conducted annually in Uganda districts, help generate timely and reliable estimates of district-level HIV prevalence.

The LQAS methodology is recommended as a tool for monitoring public health interventions [13, 14]. In Uganda, the LQAS methodology is used to monitor district-level health service interventions annually [15, 16].

Model-based SAE methods are classified into two types: 1) Unit-level models such as the Battese-Harter-Fuller model [17], which links the study or outcome variable with unit or individual-level auxiliary variables (e.g. HIV status as an outcome and individuals’ sex as the auxiliary variable) and 2) Area-level models such as the Fay-Herriot model [1], which links the study variable with summary or aggregate data of the auxiliary variable at the target geographical area (e.g., direct survey-based HIV prevalence as the outcome and percent of individuals who are women in a district as the auxiliary variable). Area-level models are applied if individual or unit-level covariate data are not available [18, 19] and are more popular than unit-level SAE methods because of the ease in accessing aggregate area-specific covariate information. Area-level models assume homogeneity of units within an area and ignore internal variability between and units within the area. Unit-level model parameters are estimated more accurately using sampling unit-level observations [1]. Unit-level models are efficient and are associated with small mean square errors (MSEs) compared to the area-level models [20].

SAE studies in Africa have been limited to area-based models to estimate institutional births in Ghana [21]; to identify unmet need for contraceptives in Ghana [22], and to estimate HIV prevalence in South Africa [23]. To our knowledge, our study is the first to use unit-level models to estimate local HIV prevalence in Uganda.

Uganda’s HIV prevalence distribution varies across geographical regions and among population groups. National HIV prevalence is 6.2% among persons aged 15–64 years (women, 7.6%; men, 4.7%) and varies from 3.1% to 8.0% across regions [24]. Availability of district-level prevalence information is therefore critical for resources allocation and decision-making.

We applied unit-level and area-level models to estimate HIV prevalence for districts in Uganda. We compared the precision of the model-based estimates to direct survey estimates and the precision of the unit-level model to the area-level model estimates. Our findings include several alternative district-level estimates that may be helpful for monitoring district-level HIV/AIDS intervention programs.

## Methods

### Data sources

#### Uganda Population HIV Impact Assessment

The Uganda Population HIV Impact Assessment (UPHIA) 2016–2017 used a two-stage, stratified cluster-sampling design. In the first stage, 520 enumeration areas (EAs) or clusters were randomly selected using probability proportional to the number of households in the cluster; in the second stage, a sample of 25 households were randomly selected using equal probabilities from each EA. The EAs were based on the 2014 National Population and Housing Census (NPHC) [25]. Uganda’s Ministry of Health conducted the survey with technical support from ICAP at Columbia University, Centers for Disease Control and Prevention, and ICF/Macro International. For detailed survey information, see the official survey report [24]. For our study, we analyzed data from 16,828 adults aged 15–64 years from 70 districts; participants provided written informed consent were tested for HIV during the survey.

#### Lot quality assurance sampling surveys

In Uganda, annual LQAS surveys are used to obtain district-level indicator estimates for monitoring health interventions [26]. In LQAS, each program area is defined to be a district subdivided into 4–7 supervision areas (SA). SA comprise either a sub-county or neighboring sub-counties with similar socioeconomic characteristics. Using probability proportional to the number of households in the village, survey staff randomly select a sample of 19 villages for districts with 5–7 SAs and 24 villages for those with four SAs. A minimum sample of 19 and 24 respondents per SA for districts with 5–7 and four SAs, respectively, are selected to maintain the combined SA misclassification (Alpha + Beta) errors to <15%. These sample sizes enable computation of district (program)-level coverage with <10% error margin for indicators [27]. A reference household is randomly identified using equal probability sampling within the SA and the next nearest household from the exit of the reference household is selected to start the survey. Eligible and consenting adult respondents are interviewed from selected households. Parallel sampling is applied to select eligible respondents for the following population categories: mothers of children aged 0–59 months, youth aged 15–24 years, women aged 15–49 years, and men aged 15–54 years. A sample of 19 or 24 respondents are selected for each of the population groups. The design does not permit selection of youth, men, and women from the same household because similar indicators are assessed in these population groups. For full details, see the survey reports [16]. We analyzed data from youth, men, and women.

#### Other sources of covariate data

Summary district level covariate data were obtained from the NPHC 2014 [28], and HIV prevalence from antenatal care attendance was obtained from the District Health Information System version 2 (DHIS2) [29]. Variables obtained from the NPHC 2014 include district population density, percentage of the population living in urban areas, and proportion of individuals who accessed a health facility in the 12 months preceding the survey.

### Statistical analysis

We computed district-level HIV prevalence estimates for 70 districts that conducted both UPHIA and LQAS surveys in 2016 using direct survey, area-level SAE models, and unit-level SAE models. We further assessed the estimates for consistency in estimating HIV prevalence and compared the precision of the estimates via the confidence intervals and the coefficient of variation of the estimates.

#### 1. Direct survey estimates

If *y*_*ij*_ is the HIV status (positive, 1; negative, 0) of the *j*-*th* individual in the *i*-*th* district and *p*_*i*_ is the proportion of HIV-positive people in district *i*, then taking into account the sampling weights, the direct estimate of district HIV prevalence is obtained as follows:

$$p_{i}=A/B$$ (1.1)

Where $A=\sum_{i}\sum_{j}w_{ij}y_{ij}$ and $B=\sum_{i}\sum_{j}w_{ij}$ and *i* = 1,2, …,*m*, *j* = 1,2, …,*N*_*i*_.

Where *m* is the number of districts and *N*_*i*_ is the number of individuals in district *i*.

Using the direct survey estimate (Eq 1.1), we used the UPHIA 2016–2017 dataset to compute weighted district HIV prevalence estimates and associated standard errors (SE) based on the sampled observation from each district. More details about the survey weights are available [24]. SE were computed using standard survey estimation (linearization) methods.

#### 2. Area-level model estimation

The multivariate Fay-Herriot model [1] is the most commonly used explicit area-level model to estimate area parameters when area-level auxiliary data are available. In area-level models, the outcome obtained from direct estimation is regressed against summary/aggregate explanatory variables that are available only at the administrative/geographical area of interest [18]. The model is defined in two stages: developing a sampling model for the direct survey estimates and applying a linking model to obtain area-level parameter estimates.

*2*.*1*. *Model estimation and prediction*. Taking $\theta_{i}=g\left({\bar{y}}_{i}\right)$, assuming g(.) is a logit link function, and relating it to a vector of *p* area-level covariates, ***z***_***i***_, where ***z***_*i*_ = (*z*_1*i*_,*z*_2*i*_, …,*z*_*pi*_) for the *m* areas, the linking model for the area-level parameter *θ*_*i*_ is defined as

$$\theta_{i}=z_{i}{}^{T}\beta +\upsilon_{i}$$ (2.1)

Where: ***β*** = (*β*_1_,*β*_2_, …,*β*_*p*_)^*T*^ is a *p* × 1 vector of regression coefficients and $\upsilon_{i}^{'}s$ are area-specific random effects assumed to be independent and identically distributed (i.i.d.) with *E*(*υ*_*i*_) = 0 and $var\;(\upsilon_{i})=\sigma_{v}^{2}\;(\text{i}.\text{e}.,\;\upsilon_{i}∼N\;(0,\sigma_{v}^{2}))$. The area-level random effects, *υ*_*i*_, capture the unstructured heterogeneity among the areas (districts) not explained by the sampling error variance.

The unbiased direct estimator of *θ*_*i*_ is obtained using a sampling model in model 2.2.

$${\hat{\theta }}_{i}=\theta_{i}+e_{i}$$ (2.2)

for *i* = 1,2, …,*m*, *e*_*i*_ ~ *N*(0,*ψ*_*i*_). Where *e*_*i*_ is the sampling error with known sampling variance, *Var* (*e*_*i*_) = *ψ*_*i*_ and *E*(*e*_*i*_) = 0 for all areas.

Model 2.2 is referred to as the sampling model because *θ*_*i*_ is unobservable and is estimated based on the sampled observations in the area.

Combining 2.1 and 2.2, we obtain the mixed model

$${\hat{\theta }}_{i}=z_{i}{}^{T}\beta +\upsilon_{i}+e_{i}$$ (2.3)

Where $\upsilon_{i}\sim N\left(0,\;\sigma_{v}^{2}\right)$, *e*_*i*_ ~ *N*(0,*ψ*_*i*_), *i* = 1,2,…,*m* and *υ*_*i*_ is independent of *e*_*i*_

The Fay-Herriot, area-level model estimate is then obtained as a weighted combination of the direct (${\hat{\theta }}_{i}$) and regression-synthetic estimators ($z_{i}{}^{T}\beta$).

$${\hat{\theta }}_{i}^{FH}={\hat{\gamma }}_{i}{\hat{\theta }}_{i}+\left(1-{\hat{\gamma }}_{i}\right)z_{i}{}^{T}\beta$$ (2.4)

Where: ${\hat{\gamma }}_{i}=\frac{{\hat{\sigma }}_{v}^{2}}{{\hat{\sigma }}_{v}^{2}+\sigma_{i}^{2}}$

The weighting component is the ratio of the model error variance to the total variance. From Eq 2.4, the estimate ${\hat{\theta }}_{i}^{FH}$ tends to ${\hat{\theta }}_{i}$ for large values of the model variance (${\hat{\sigma }}_{v}^{2}$) and tends to $z_{i}{}^{T}\beta$ for small values of the model variance relative to $\sigma_{i}^{2}$. Model parameters and SE are estimated using maximum likelihood methods [18, 30].

*2*.*2*. *Auxiliary variables for the area-level model*. Variants of the area-level model were fitted with different combinations of the predictor variables as shown in S1 File. The general population direct HIV prevalence estimate (*p*_*i*_) was found to be related to HIV prevalence from ANC attendance (*P*_*ANCi*_) *y*_*i*_ = *logit*(*p*_*i*_) and ***z***_*i*_ = *logit*(*p*_*ANCi*_). This model had the lowest value of the Akaike Information Criterion (AIC) (Table 1 in S1 File). The models were fitted using the SAE package in R version 3.6.2 [31].

#### 3. Unit-level model estimation

When unit-level or individual-level auxiliary data $X_{ij}=\left(x_{ij1},\;x_{ij2},\;,x_{ij3},\ldots .x_{ijq},\right)$ for a vector of *q* auxiliary variables are available, the Battese-Harter-Fuller [17] unit-level model is often applied to obtain small area parameter estimates. Under the unit-level model, data for both the outcome and the explanatory variables are available for each population element in the district, irrespective of the administrative area/domain of interest [18].

*3*.*1*. *Model estimation and prediction*. Letting *p*_*ij*_ be the probability of individual *j* from district *i* being HIV positive ($p_{ij}=Pr\text{(}y_{ij}=1\;|\;x_{ij},\;\mu_{i})$, where *y*_*ij*_ corresponds to the HIV status of individual *i* in district, a logistic regression model with area-level effect is used to estimate *p*_*i*_ [18, 32].

$$\text{logit}\left(p_{ij}\right)=\log\left(\frac{p_{ij}}{1-p_{ij}}\right)=X_{ij}^{t}\beta +\upsilon_{i}+e_{ij}$$ (3.1)

Where *y*_*ij*_ is assumed to be independent Bernoulli (*p*_*ij*_) conditioned on *p*_*ij*_’s with area random effects *υ*_*i*_; *β* is the vector of regression model parameters. *υ*_*i*_ is assumed to be independent and identically distributed with *E*(*υ*_*i*_) = 0 and $Var\;(\upsilon_{i})=\sigma_{v}^{2}$.

Assuming absence of area-level auxiliary information, the indirect estimators of district HIV prevalence, based on model 3.1 is the empirical best predictor obtained as follows:

$${\hat{p}}_{i}=\frac{1}{N_{i}}\left\{\sum_{j\in S}y_{ij}+\sum_{j\in S^{\prime }}{\hat{p}}_{ij}\right\}$$ (3.2)

Where ${\hat{p}}_{ij}=\frac{exp\left({\hat{\eta }}_{ij}\right)}{1+exp\left({\hat{\eta }}_{ij}\right)}$ and ${\hat{\eta }}_{ij}={\hat{\beta }}_{i}X^{t}{}_{ij}+{\hat{v}}_{i}$

The component $\sum_{j\in S}y_{ij}$, is the sum of *n*_*i*_ values of HIV infection for sampled individuals from the *i*-*th* district while $\sum_{j\in S^{\prime }}{\hat{p}}_{ij}$ is the sum over the estimated probability of infection for the non-sampled individuals in district *i*, and *N*_*i*_ corresponds to number of individuals in each district. Model fitting and parameter estimation were implemented using the SAE [31] package in R software, version 3.6.2 [31]

The MSE of ${\hat{p}}_{i}$ is obtained using the parametric bootstrap estimation method for finite populations [33, 34] as described in S2 File.

*3*.*2*. *Auxiliary variables for the unit-level model*. Auxiliary variables from the 2016 LQAS data include age group in years (15–19, 20–24, 25–34, 35–44, and ≥45), sex (male and female), level of education (none, primary, secondary, and tertiary), marital status (single, married, and previously married: widowed/divorced/separated) and number of sexual partners including spouse in the 12 months preceding the survey (0, 1, and ≥2). We selected these variables because they were significantly associated with HIV positivity in our previous study [6].

#### 4. Comparison of the unit-level and area-level model estimates

We used summary statistics, regression analysis, and graphical assessment for consistency to compare estimates from direct, area-level, and unit-level models. We assessed gain in precision of the model-based estimates compared to the direct survey estimates using the coefficient of variation of the estimate and ratio of the MSE. We further computed ratios of the unit-level model and the area-level model estimates to assess for improvement in precision of the unit-level model estimates.

### Ethics approval and consent to participate

Ethical clearance to conduct this study was obtained from the University of Witwatersrand Human Research Ethics Committee (HREC), clearance Certificate number M171053. Further clearance was obtained from the Uganda National Council for Science and Technology (UNCST) with registration number HS2366. Data for the study were obtained from surveys conducted in Uganda. The study was a secondary analysis of data, so consent to participate is not applicable.

## Results

### Selected characteristics of survey participants

For both surveys, most respondents were women, had incomplete primary education, were married/cohabiting, and had one sexual partner in the 12 months preceding the surveys (Table 1).

**Table 1:** Characteristics of respondents.

	UPHIA 2016	LQAS 2016
Characteristic	Weighted % (n = 16,862)	%(n = 34,109)
**Sex**		
Male	47.9 (7,302)	48.5 (16,545)
Female	52.1 (9,560)	51.5 (17,562)
**Age, years**		
15–19	23.9 (3,649)	23.5 (7,997)
20–24	18.6 (2,859)	24.6 (8,403)
25–34	25.2 (4,190)	23.3 (7,946)
35–44	16.3 (2,885)	18.8 (6,407)
45–64	14.4 (3,279)	9.8 (3,356)
**Education level#**		
No education	7.8 (1,582)	7.4 (2,530)
Incomplete primary	43.7 (7,663)	42.6 (14,538)
Primary	16.2 (2,604)	23.2 (7,905)
Incomplete secondary	24.0 (3,695)	17.1 (5,845)
Complete secondary+	8.3 (1,218)	9.7 (3,291)
**Marital status##**		
Never married	31.1 (4,620)	33.5 (11,435)
Married/cohabiting	55.7 (9,775)	61.6 (21,022)
Widowed	3.5 (705)	1.5 (524)
Separated	9.8 (1,728)	3.3 (1,127)
**Number of sexual partners in last 12 months###**		
0	14.1 (2,131)	31.5 (9,723)
1	70.2 (10,261)	57.5 (17,760)
≥2	15.6 (2,125)	11.0 (3,410)

Notes: UPHIA 2016 survey data weighted using the population survey weight. Abbreviations: UPHIA, Uganda Population-Based HIV Impact Assessment; LQAS, Lot Quality Assurance Surveys. Data missing for #-101, ##-39 and ###-2345 respondents.

### HIV prevalence estimates

HIV prevalence estimates for the districts included in the analysis are summarized in Table 2. District-specific estimates with 95% confidence intervals are presented in S1 Table. On average, direct survey estimates were higher (mean = 0.064, SD = 0.034) than area-level (mean = 0.056, SD = 0.018) and unit-level model (mean = 0.058, SD = 0.026) estimates. Direct survey estimates also had a higher variation than the model-based estimates, with minimum and maximum values of 0.010 and 0.148, respectively. The estimates from the area-level model had the least variation (Table 2).

**Table 2:** Summary of district-level HIV prevalence estimates in Uganda.

	Mean (SD)	Minimum	Maximum	First Quartile (Q1)	Second Quartile (Q2)	Third Quartile (Q3)
Direct survey	0.064 (0.034)	0.010	0.148	0.038	0.055	0.089
Area-level model	0.056 (0.018)	0.0157	0.096	0.042	0.058	0.067
Unit-level model	0.058 (0.026)	0.004	0.142	0.042	0.042	0.074

**Fig 1:**
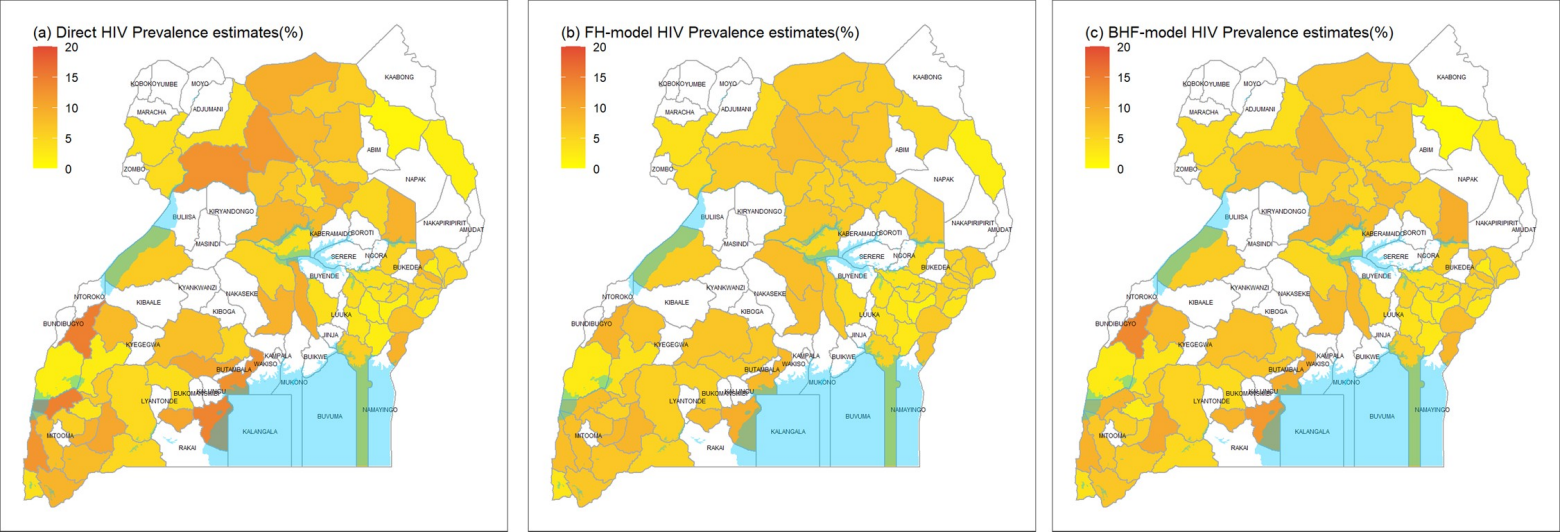
Comparison of HIV prevalence estimates in Uganda. Fig 1 presents district-level prevalence estimates: (a) Direct survey estimates, (b) area-level model estimates, and (c) unit-level model estimates. Scales for each of the maps were maintained to show the extent of extreme values. Unshaded areas represent district that did not complete LQAS surveys in 2016.

Unit-level model HIV prevalence estimates Fig 1C had a similar pattern compared to the direct survey prevalence estimates (Fig 1A). HIV prevalence estimates based on the area-level model had the least recognizable pattern between districts (Fig 1B) consistent with the summary statistics in Table 2. HIV prevalence was generally higher in districts in Central, South Western, and Northern regions of Uganda and in districts bordering lakes.

### Model diagnostics

**Fig 2:**
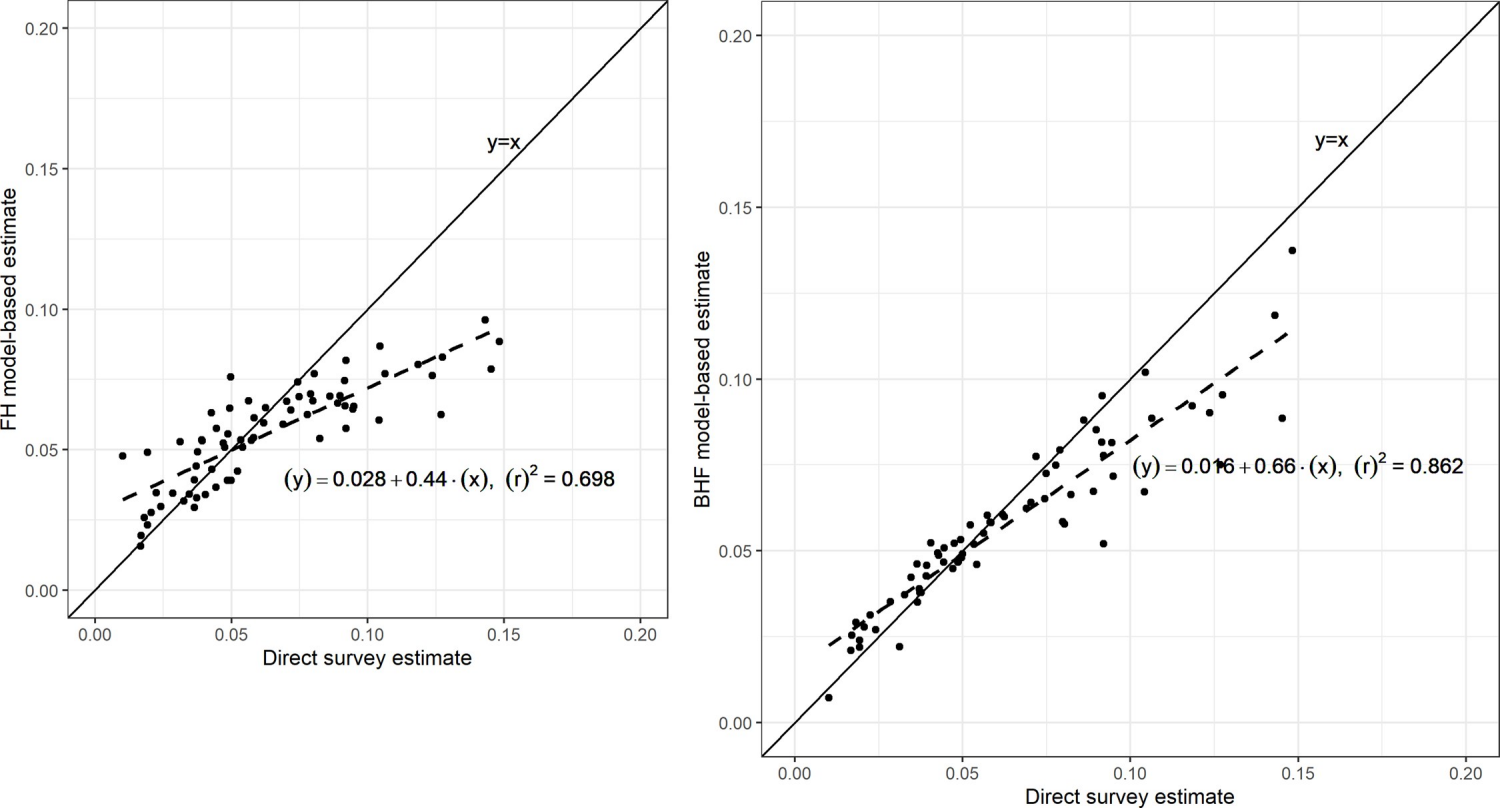
Correlation of model and direct survey estimates of HIV prevalence in Uganda. Regression and scatter plot of (a) area-level model estimates compared to direct survey estimates and (b) unit-level model estimates compared to direct survey estimates.

We regressed model-based estimates against direct survey estimates to assess bias and reliability of the model-based estimates. The bias diagnostics plot also presents a scatter plot of the estimates and shows the effect of extreme values in the estimates. Unit-level model estimates were strongly correlated with the direct survey estimates (*β*_1_ = 0.66; *r*^2^ = 0.862; Fig 2B) compared to area-level model estimates (*β*_1_ = 0.44; *r*^2^ = 0.698; Fig 2A).

### Precision and consistency of HIV prevalence estimates

**Fig 3:**
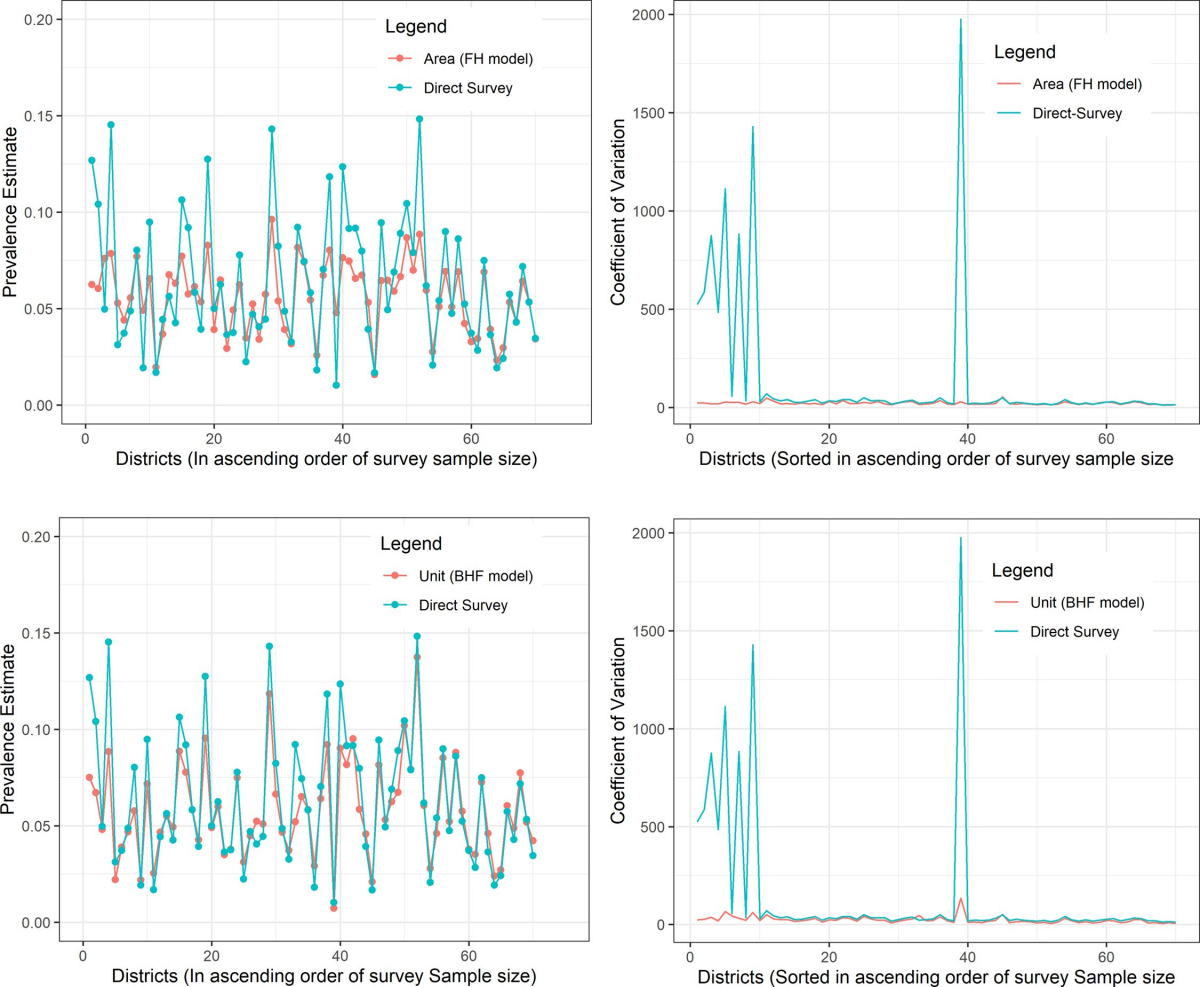
HIV prevalence estimates and the coefficient of variation of direct survey and model-based estimates in Uganda. HIV prevalence estimates (on the left) and the coefficient of variation (on the right) for direct survey and model-based estimates with the districts sorted in ascending order of the survey sample sizes. (a) direct survey compared to area-level model estimates using the Fay-Herriot (FH) model, (b) coefficient of variation for direct survey and area-level model estimates, (c) direct survey compared to unit-level model estimates using the Battese-Harter-Fuller (BHF) model, and (d) coefficient of variation for direct survey and unit-level model estimates.

The model-based estimates were similar to the direct survey estimates, but the point estimates had less variation compared to the direct survey estimates (Fig 3A and 3C). We also note that direct survey estimates were significantly different (i.e., they were either higher or lower) compared with model-based estimates for small survey sample sizes Fig 3A and 3C), demonstrating the shrinkage of the model-based estimates toward the point estimates. However, direct survey and model-based estimates tended to be similar with increasing survey sample sizes in the districts. For example, Nwoya district with survey sample of 44 individuals, the HIV prevalence estimate was 0.127 (95% CI: 0.000–1.000), 0.062 (95% CI: 0.034–0.091), and 0.075 (95% CI: 0.039–0.111), whereas for Mbale district with a survey sample of 874 individuals, the estimates were 0.054 (95% CI, 0.028), 0.051 (95% CI: 0.029, 0.072), and 0.046 (95% CI: 0.029, 0.063) for direct survey, area-level and unit-level models respectively (S1 Table). The average improvement in the precision of the estimates was 37.5% and 33.1% for the unit-level and area-level model, respectively (data not shown).

The coefficient of variation of the direct survey estimates were generally larger and had a higher variation compared to the coefficient of variation of the model-based estimates irrespective of the survey sample size in the districts (Fig 3B and 3D). Table 3 summarizes the coefficients of variation and shows that the mean coefficient of variation for direct survey estimates was 138.0% (346.9) compared to 22.4% (7.5) for the area-level and 23.7% (18.5) unit-level models.

**Table 3:** Summary of coefficient of variation of HIV prevalence estimates in Uganda*.

	Mean (SD)	Minimum	Maximum	First Quartile (Q1)	Second Quartile (Q2)	Third Quartile (Q3)
Direct survey	138.0 (346.9)	13.3	1974.8	21.9	28.0	40.2
Area-level model	22.4(7.5)	13.3	54.6	17.5	19.9	19.7
Unit-level model	23.7 (18.5)	5.6	133.8	12.4	19.7	27.7

*Omitting the outlier district (Kalangala) from the analysis did not affect the model-based or direct survey estimates (results not presented). Abbreviations: SD, Standard Deviation.

Assuming estimates are considered as reliable for decision making if the coefficient of variation is <20% [31], then <50% of the districts have reliable data for decision making based on the direct survey estimates, whereas >50% of the districts would have reliable data based on the SAE methods (Table 3). Specifically, only 14 (20%), 36 (51.4%), and 36 (51.4%) of the districts would have reliable information for decision making based on direct survey estimates, area-level model estimates, and unit-level model estimates, respectively (data not shown).

### Consistency of model-based estimates

**Fig 4:**
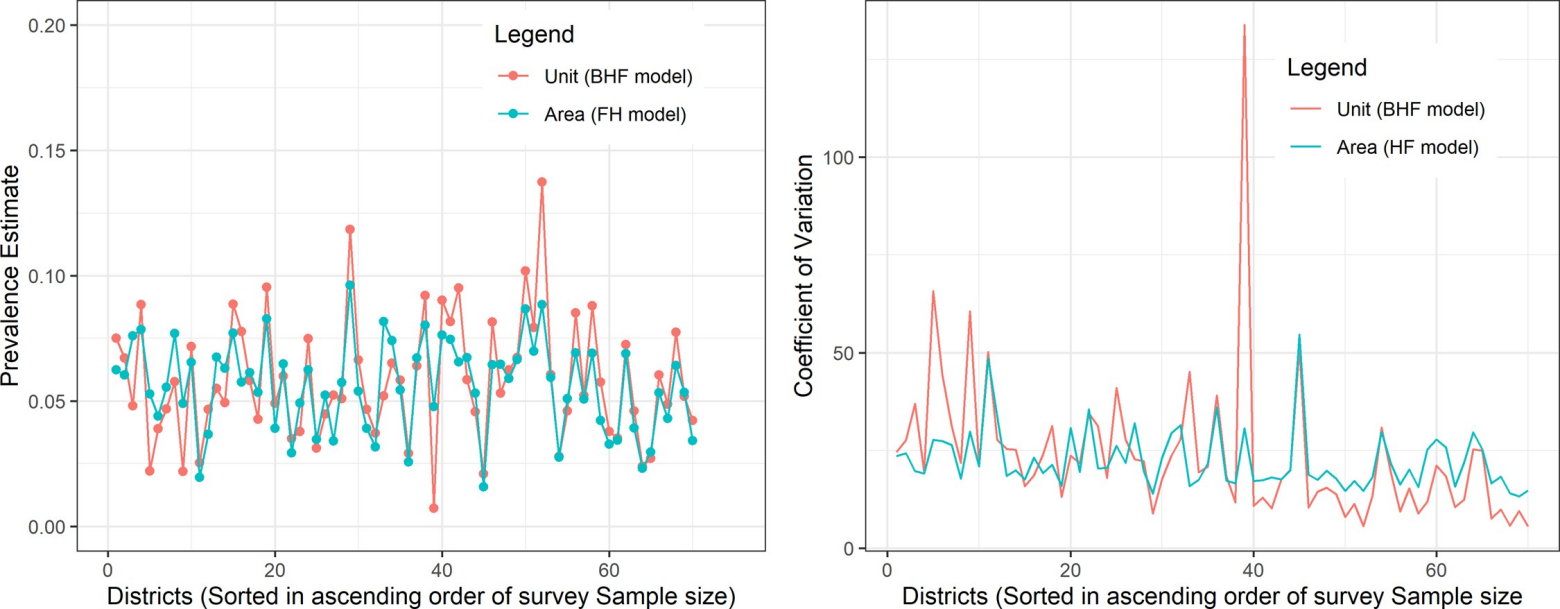
Area-level and unit-level model-based estimates of district HIV prevalence in Uganda. (a) HIV prevalence estimates and (b) the coefficient of variation for area-level and unit-level model-based estimates with the districts sorted in ascending order of the survey sample sizes.

Unit-level model estimates varied more than the area-level model estimates (Fig 4A). Additionally, the coefficients of variation of the unit-level model estimates were consistently larger for districts with small survey sample sizes and consistently smaller for districts with large survey sample sizes (Fig 4B). Implying that the unit-level model estimates converge more rapidly to the point estimate compared to the area-level model estimates as the survey sample sizes in the districts increased.

## Discussion

Study findings show the feasibility of applying SAE models to population survey data with auxiliary information from community LQAS surveys and DHIS2 data to obtain more precise HIV prevalence estimates for districts in Uganda. Both the unit-level and area-level model estimates were similar to the direct survey estimates, although the unit-level model estimates were more correlated with the direct survey estimates compared to area-level model estimates. A graphical assessment shows that the model-based estimates were close to the direct survey estimates, were less polarized, and had no extreme values/outliers. Mapping model-based HIV prevalence estimates shows a similar pattern between the unit-level estimates and the direct survey estimates. Estimates for all approaches were generally similar to regional survey prevalence estimates [24].

The coefficient of variation of both the unit-level and area-level model estimates were lower than the coefficient of variation of the direct survey estimates. However, the coefficient of variation of the unit-level model estimates were larger than the coefficient of variation of the area-level model estimates, which suggests that area-level model estimates were more precise. Coefficient of variation is computed as a ratio of the SE to the mean and expressed as a percentage. It therefore expresses the sampling variability of the estimates from the point estimate, implying that estimates with large coefficients of variation would be considered over-dispersed and therefore unreliable for decision-making.

Although population surveys provide more accurate national and regional information, they yield only partial information for district-level planning and allocating resources. Uganda, like many other low and middle-income countries, lacks the resources to collect representative data for monitoring social services at the district-level; however, in Uganda’s de-centralized governance model, districts plan, implement, and monitor interventions on behalf of the central government. Using simple methods to generate more precise HIV prevalence estimates for districts will therefore augment district-level decision making. Application of SAE methods elsewhere in Africa have shown more precise district-level estimates [21–23] as observed in our study.

We note that the unit-level model estimates were highly correlated with direct survey estimates, although they were less precise compared to the area-level model estimates, which is contrary to SAE’s methodological theory [18]. Lower correlation of the area-level model estimates with direct survey estimates compared to the unit-level model estimates versus direct survey estimates may be attributed to the aggregated area-level covariates, which mask any internal variations or heterogeneity of units. The heterogeneous spread of HIV is well documented in literature [6, 23, 24]. For example, HIV prevalence is higher in urban areas compared to rural areas [6, 23, 24]. Similarly, females have a higher HIV positivity compared to males although the differences between males and females varies by age group [24]. The difference is wider for the younger age group 15–24 years and converges with increasing age [24]. It is therefore important to take into consideration, this internal differences in obtaining the estimates as demonstrated by the BHF model applied in this study.

Additionally, use of antenatal data for HIV prevalence monitoring in the general population has limitations and biases including selection bias of routine data. Antenatal HIV surveillance data excludes non-pregnant women and men and includes information from health facilities in urban and easily accessible areas [35]; public health facilities that report to national HMIS system [20]; younger and more educated women who have higher antenatal care attendance rates [36–38]. These limitations imply that the antenatal survey data may not accurately reflect the general population HIV prevalence distribution as observed in our study.

Overall improvement in the precision of the estimates was 37.5% and 33.1% for the unit-level and area-level model, respectively. A study combining population survey with routine data found 28% improvement in the precision of estimates but lower correlation with estimates based on routine data [5]. Use of risk factor data, representative of the general population, therefore improves the precision of the estimates compared to combining routine and survey data or use of more aggregate information.

Study findings were consistent with regional HIV prevalence estimates based on survey data. Districts with higher HIV prevalence were from regions with overall higher HIV prevalence (e.g., Kaborale district in Western region; Masaka and Mpigi in central 1 region; and Mbarara in South Western region) as observed in the national level survey [24]. These districts are urban and are major transport corridors for truck drivers. Masaka district also is inhabited by fishing communities, which typically have higher HIV prevalence [8, 9, 39, 40].

Although the model-based methods assume independence of area random effects, independence is unlikely to hold between neighboring districts. Spatial estimation approaches attempt to solve this, although these methods have their own limitations [23]. Borders and boundaries are arbitrary, and individuals tend to seek healthcare services in neighboring districts or even other regions that are convenient or offer better quality services. For example, some districts in Uganda do not have Health Centre IV or hospitals, which are known to provide better quality healthcare and a broad range of health services [23]. Furthermore, 40 out of 112 districts in the country did not conduct LQAS surveys in 2016; this implies that BHF model estimates could not be obtained for these districts limiting the breath of model evaluation.

Data-driven district-level decision making for HIV programs requires precise and reliable estimates, but survey sample sizes from population surveys are significantly smaller for districts than for regions. Our study shows that with external auxiliary data, estimates that are more precise can be obtained, but precision depends on whether the information is available at the sampling unit level or area level. Unit-level model estimates, although less precise than area-level estimates, were more consistent with direct survey estimates. This application also promotes use of annual community LQAS data, which is readily available to district service managers. SAE models also can be developed in freely available software, such as R and STATA. Models that use both area-level and unit-level covariates to obtain SAE could help provide more precise and accurate HIV prevalence estimates in other settings.

## Supporting information

S1 File Area-level model of HIV prevalence in Uganda. (DOCX)

S2 File Parametric bootstrap for mean square error estimation for Battese-Harter-Fuller (BHF) model. (DOCX)

S1 Table District HIV prevalence estimates in Uganda using direct estimates, Fay-Herriot model estimates, and Battese-Harter-Fuller model estimates. Abbreviations: CI, confidence interval.
(DOCX)
