# Supplementary material for: Model-based small area estimation methods and precise district-level HIV prevalence estimates in Uganda
Source: PLoS One. 2021 Aug 6;16(8):e0253375. doi: 10.1371/journal.pone.0253375 (PMC8345831; doi:10.1371/journal.pone.0253375)
Supplement: S1 File — (DOCX) [file pone.0253375.s001.docx]

**S1 File: Area-level model of HIV prevalence in Uganda**

District-level covariates were obtained from 2014 National Population and Housing Census (NPHC) data and 2016 District Health Information System, version 2 (DHIS2) data. Variants of the basic Fay-Herriot (FH) area-level model were fitted with different combinations of the predictor variables. The final small area estimation (SAE) model was chosen as that with the lowest Akaike Information Criterion (AIC). The models were fitted using the SAE package in R version 3.6.2 [30]. The variables assessed were district population density (Pop density), percent of the population living in urban areas (Urban Pct), proportion of individuals who accessed a health facility in the 12 months preceding the survey (HF utilization), and antenatal HIV prevalence (ANC) from DHIS2 data. We assumed that the general population direct HIV prevalence estimate ($p_{i})$ is related to HIV prevalence from ANC attendance $\left( p_{ANCi} \right)$ $y_{i}=logit\left( p_{i} \right)$ and $\boldsymbol{z}_{i}=logit\left( p_{ANCi} \right)$. The final model was fitted with the log of ANC prevalence as the predictor to obtain the FH area-level HIV prevalence estimates and mean square error (MSE) $\boldsymbol{z}_{i}$ as the only predictor using the SAE[30] package in R[41].

**Table 1. Variable selection of the FH model**

| **Model** | **Predictors** | **Log likelihood** | **AIC** |
| --- | --- | --- | --- |
| 1 | ANC | 220.74 | -435.49 |
| 2 | Log(ANC) | 222.64 | -439.29 |
| 3 | Pop density | 196.27 | -386.54 |
| 4 | Urban Pct | 198.30 | -390.59 |
| 5 | HF utilization | 197.31 | -388.61 |
| 6 | Log(ANC) + Urban Pct | 223.15 | -438.30 |
| 7 | Log(ANC) + Urban + HF Utilization + Pop density | 223.29 | -434.59 |
| 8 | Urban+HF Utilization + Pop density | 200.77 | -391.53 |
