# Supplementary material for: Model-based small area estimation methods and precise district-level HIV prevalence estimates in Uganda
Source: PLoS One. 2021 Aug 6;16(8):e0253375. doi: 10.1371/journal.pone.0253375 (PMC8345831; doi:10.1371/journal.pone.0253375)
Supplement: S2 File — (DOCX) [file pone.0253375.s002.docx]

**S2 File. Parametric Bootstrap for Mean Square Error estimation for Battese-Harter-Fuller (BHF) model**

Estimation of the mean square error (MSE) of the Empirical Bayes (EB) estimates for the unit-level model is obtained using the parametric bootstrap for finite population:

1. Fit the basic unit-level model by Maximum likelihood or Restricted Maximum Likelihood to obtain model parameters $\hat{\beta}^{*}$, $\sigma_{v}^{2}$ and $\sigma_{i}^{2}$
2. Generate bootstrap domain effects assumed to beindependent and identically distributed (i.i.d.) with $E\left( \upsilon_{i} \right)=0$ and $var \left( \upsilon_{i} \right)=\sigma_{v}^{2}$ (i.e., $\upsilon_{i}\sim N\left( 0, \sigma_{v}^{2} \right)$).
3. Generate, independent of the domain effects, $\upsilon_{i}$ unit errors as

$e_{ij}\sim N\left( 0, \sigma_{e}^{2} \right)$, for $i$= 1, 2, …$m$ for $j$= 1, 2, …$N$

1. Generate a bootstrap population of response variables from the model

$$y_{ij}^{*}=X_{ij}^{t}\beta+\upsilon_{i}+e_{ij}$$

2. Letting $y_{i}^{p*}=\left( y_{i1}^{*}, y_{i2}^{*},\ldots y_{iN}^{*} \right)^{T}$ denote the vector of generated bootstrap response variables for area $i$, We calculate the target quantities for the bootstrap population as

$$\tau_{i}^{*}=h\left( y_{i}^{p*} \right), i=1,2,\ldots m$$

3. Letting $y_{s}^{*}$ be the vector whose elements are the generated $y_{ij}^{*}$ with indices contained in the sample, fit the model to the bootstrap sample data $\left( y_{ij}^{*}, X_{ij} \right);J\in S_{i}, i=1,2,\ldots,m$ to obtain bootstrap model parameter estimators, denoted by $\hat{\beta}^{*}$, $\sigma_{v}^{2*}$ and $\sigma_{i}^{2*}$
4. Obtain the bootstrap EB estimator of $\tau_{i}$through the Monte-Carlo approximation, denoted by $\hat{\tau}_{i}^{EB*}, i=1,2,\ldots m$
5. Repeat steps 2–7 many times, (B). let $\hat{\tau}_{i}^{*}\left( b \right)$ be the true value and $\hat{\tau}_{i}^{EB*}\left( b \right)$ the EB estimator obtained in the $b^{th}$-replicate of the bootstrap procedure, $b=1,2,\ldots, B$. Where B is the number of replications/simulations
6. The bootstrap MSE estimator of $\hat{\tau}_{i}^{EB}$ is given by

$${mse}_{B}\left( \hat{\tau}_{i}^{EB} \right)=B^{-1}\sum_{b=1}^{B} \left[ \hat{\tau}_{i}^{EB*}\left( b \right)-\tau_{i}^{*}\left( b \right) \right]^{2}$$
