# Supplementary material for: Model-based small area estimation methods and precise district-level HIV prevalence estimates in Uganda
Source: PLoS One. 2021 Aug 6;16(8):e0253375. doi: 10.1371/journal.pone.0253375 (PMC8345831; doi:10.1371/journal.pone.0253375)
Supplement: S1 Table — Abbreviations: CI, confidence interval. (DOCX) [file pone.0253375.s003.docx]

**S1 Table. District HIV prevalence estimates in Uganda using direct estimates, Fay-Herriot model estimates, and Battese-Harter-Fuller model estimates**

| **Region/**  **District** | **Survey Sample** | **Direct survey estimates**  **( 95% CI)** | | **FH model estimates**  **(95% CI)** | | | | | **BHF model estimates**  **(95% CI)** | | | |
| --- | --- | --- | --- | --- | --- | --- | --- | --- | --- | --- | --- | --- |
| **Central 1** |  |  |  | |  |  |  |  |  |  |  |  |
| Gomba | 46 | 0.104 (0.000 – 1.000) | | 0.060 (0.032 - 0.089) | | | | | 0.067 (0.031 - 0.103) | | | |
| Lwengo | 131 | 0.127 (0.073 - 0.182) | | 0.083 (0.057 - 0.109) | | | | | 0.095 (0.071 - 0.120) | | | |
| Masaka | 176 | 0.104 (0.000 - 1.000) | | 0.060 (0.032 - 0.089) | | | | | 0.067 (0.031 - 0.103) | | | |
| Mpigi | 139 | 0.106 (0.052 - 0.161) | | 0.077 (0.050 - 0.104) | | | | | 0.089 (0.061 - 0.116) | | | |
| Ssembabule | 135 | 0.127 (0.073 - 0.182) | | 0.083 (0.057 - 0.109) | | | | | 0.095 (0.071 - 0.120) | | | |
| **Central 2** |  |  |  | |  |  |  |  |  |  |  |  |
| Kayunga | 233 | 0.094 (0.057 - 0.132) | | 0.064 (0.041 - 0.088) | | | | | 0.081 (0.065 - 0.098) | | | |
| Luwero | 199 | 0.075 (0.047 - 0.102) | | 0.069 (0.048 - 0.090) | | | | | 0.073 (0.058 - 0.088) | | | |
| Mityana | 233 | 0.050 (0.000 - 0.902) | | 0.076 (0.047 - 0.105) | | | | | 0.048 (0.013 - 0.083) | | | |
| Mubende | 346 | 0.094 (0.057 - 0.132) | | 0.064 (0.041 - 0.088) | | | | | 0.081 (0.065 - 0.098) | | | |
| Nakasongola | 53 | 0.075 (0.047 - 0.102) | | 0.069 (0.048 - 0.090) | | | | | 0.073 (0.058 - 0.088) | | | |
| **East Central** |  |  |  | |  |  |  |  |  |  |  |  |
| Bugiri | 292 | 0.021 (0.004 - 0.037) | | 0.028 (0.011 - 0.044) | | | | | 0.028 (0.011 - 0.045) | | | |
| Busia | 324 | 0.090 (0.060 - 0.120) | | 0.069 (0.047 - 0.091) | | | | | 0.085 (0.069 - 0.101) | | | |
| Iganga | 343 | 0.041 (0.012 - 0.069) | | 0.034 (0.013 - 0.055) | | | | | 0.052 (0.029 - 0.076) | | | |
| Kaliro | 169 | 0.057 (0.036 - 0.079) | | 0.053 (0.036 - 0.071) | | | | | 0.060 (0.051 - 0.069) | | | |
| Kamuli | 366 | 0.018 (0.000 - 0.036) | | 0.026 (0.008 - 0.044) | | | | | 0.029 (0.007 - 0.052) | | | |
| Mayuge | 495 | 0.090 (0.060 - 0.120) | | 0.069 (0.047 - 0.091) | | | | | 0.085 (0.069 - 0.101) | | | |
| Namutumba | 184 | 0.041 (0.012 - 0.069) | | 0.034 (0.013 - 0.055) | | | | | 0.052 (0.029 - 0.076) | | | |
| **Eastern** |  |  |  | |  |  |  |  |  |  |  |  |
| Amuria | 333 | 0.047 (0.025 - 0.070) | | 0.051 (0.031 - 0.071) | | | | | 0.052 (0.037 - 0.068) | | | |
| Budaka | 146 | 0.050 (0.016 - 0.084) | | 0.039 (0.016 - 0.063) | | | | | 0.049 (0.026 - 0.072) | | | |
| Bududa | 179 | 0.049 (0.017 - 0.080) | | 0.039 (0.017 - 0.062) | | | | | 0.047 (0.025 - 0.068) | | | |
| Bukwo | 110 | 0.082 (0.042 - 0.123) | | 0.054 (0.029 - 0.078) | | | | | 0.066 (0.044 - 0.089) | | | |
| Bulambuli | 177 | 0.017 (0.000 - 0.033) | | 0.016 (0.000 - 0.032) | | | | | 0.021 (0.000 - 0.042) | | | |
| Butaleja | 227 | 0.019 (0.000 - 0.558) | | 0.049 (0.020 - 0.078) | | | | | 0.022 (0.000 - 0.048) | | | |
| Kapchorwa | 100 | 0.033 (0.009 - 0.057) | | 0.032 (0.012 - 0.051) | | | | | 0.037 (0.017 - 0.057) | | | |
| Katakwi | 222 | 0.052 (0.030 - 0.075) | | 0.042 (0.021 - 0.063) | | | | | 0.058 (0.044 - 0.071) | | | |
| Kibuku | 179 | 0.043 (0.009 - 0.076) | | 0.063 (0.038 - 0.088) | | | | | 0.049 (0.025 - 0.074) | | | |
| Kumi | 336 | 0.024 (0.010 - 0.038) | | 0.030 (0.015 - 0.044) | | | | | 0.027 (0.014 - 0.040) | | | |
| Kween | 130 | 0.053 (0.039 - 0.068) | | 0.053 (0.040 - 0.067) | | | | | 0.052 (0.042 - 0.062) | | | |
| Manafwa | 448 | 0.037 (0.017 - 0.057) | | 0.033 (0.015 - 0.051) | | | | | 0.038 (0.022 - 0.054) | | | |
| Mbale | 874 | 0.054 (0.028 - 0.080) | | 0.051 (0.029 - 0.072) | | | | | 0.046 (0.029 - 0.063) | | | |
| Pallisa | 337 | 0.047 (0.025 - 0.070) | | 0.051 (0.031 - 0.071) | | | | | 0.052 (0.037 - 0.068) | | | |
| Sironko | 298 | 0.049 (0.017 - 0.080) | | 0.030 (0.017 - 0.062) | | | | | 0.047 (0.025 - 0.068) | | | |
| Tororo | 685 | 0.082 (0.042 - 0.123) | | 0.054 (0.029 - 0.078) | | | | | 0.066 (0.044 - 0.089) | | | |
| **Karamoja** |  |  |  | |  |  |  |  |  |  |  |  |
| Kotido | 193 | 0.010 (0.000 - 0.403) | | 0.048 (0.019 - 0.076) | | | | | 0.007 (0.000 - 0.026) | | | |
| Moroto | 108 | 0.017 (0.000 - 0.040) | | 0.019 (0.001 - 0.038) | | | | | 0.025 (0.000 - 0.050) | | | |
| **Northern** |  |  |  | |  |  |  |  |  |  |  |  |
| Agago | 191 | 0.070 (0.036 - 0.105) | | 0.067 (0.044 - 0.090) | | | | | 0.064 (0.041 - 0.087 | | | |
| Alebtong | 133 | 0.092 (0.044 - 0.140) | | 0.058 (0.031 - 0.084) | | | | | 0.078 (0.049 - 0.106) | | | |
| Amolatar | 150 | 0.038 (0.008 - 0.067) | | 0.049 (0.030 - 0.069) | | | | | 0.038 (0.015 - 0.061) | | | |
| Amuru | 68 | 0.037 (0.000 - 0.079) | | 0.044 (0.020 - 0.068) | | | | | 0.039 (0.005 - 0.073) | | | |
| Apac | 336 | 0.086 (0.057 - 0.115) | | 0.069 (0.048 - 0.090) | | | | | 0.088 (0.073 - 0.103) | | | |
| Dokolo | 150 | 0.078 (0.037 - 0.119) | | 0.062 (0.037 - 0.088) | | | | | 0.075 (0.048 - 0.101) | | | |
| Gulu | 191 | 0.118 (0.073 - 0.163) | | 0.080 (0.054 - 0.106) | | | | | 0.092 (0.071 - 0.113) | | | |
| Kitgum | 134 | 0.058 (0.021 - 0.096) | | 0.061 (0.038 - 0.084) | | | | | 0.058 (0.031 - 0.086) | | | |
| Kole | 184 | 0.058 (0.026 - 0.091) | | 0.054 (0.031 - 0.077) | | | | | 0.058 (0.035 - 0.082) | | | |
| Lamwo | 101 | 0.095 (0.038 - 0.151) | | 0.065 (0.039 - 0.092) | | | | | 0.072 (0.042 - 0.102) | | | |
| Lira | 226 | 0.039 (0.015 - 0.063) | | 0.053 (0.032 - 0.074) | | | | | 0.046 (0.028 - 0.064) | | | |
| Nwoya | 44 | 0.127 (0.000 - 1.000) | | 0.062 (0.034 - 0.091) | | | | | 0.075 (0.039 - 0.111) | | | |
| Otuke | 87 | 0.049 (0.000 - 0.893) | | 0.056 (0.027 - 0.084) | | | | | 0.047 (0.018 - 0.075) | | | |
| Oyam | 243 | 0.069 (0.038 - 0.100) | | 0.059 (0.036 - 0.082) | | | | | 0.062 (0.043 - 0.081) | | | |
| Pader | 97 | 0.080 (0.026 - 0.134) | | 0.077 (0.050 - 0.104) | | | | | 0.058 (0.033 - 0.083) | | | |
| **South Western** |  |  |  | |  |  |  |  |  |  |  |  |
| Buhweju | 60 | 0.031 (0.000 - 0.713) | | 0.053 (0.024 - 0.081) | | | | | 0.022 (0.000 - 0.051) | | | |
| Bushenyi | 128 | 0.056 (0.018 - 0.095) | | 0.067 (0.043 - 0.092) | | | | | 0.055 (0.028 - 0.082) | | | |
| Ibanda | 147 | 0.062 (0.025 - 0.100) | | 0.065 (0.040 - 0.090) | | | | | 0.060 (0.034 - 0.086) | | | |
| Isingiro | 166 | 0.047 (0.015 - 0.079) | | 0.052 (0.030 - 0.075) | | | | | 0.045 (0.020 - 0.069) | | | |
| Kabale | 258 | 0.089 (0.054 - 0.124) | | 0.067 (0.043 - 0.090) | | | | | 0.067 (0.049 - 0.086) | | | |
| Kanungu | 199 | 0.124 (0.075 - 0.172) | | 0.076 (0.051 - 0.102) | | | | | 0.090 (0.071 - 0.109) | | | |
| Kiruhura | 169 | 0.044 (0.014 - 0.075) | | 0.057 (0.035 - 0.080) | | | | | 0.051 (0.029 - 0.073) | | | |
| Kisoro | 149 | 0.036 (0.008 - 0.065) | | 0.029 (0.009 - 0.050) | | | | | 0.035 (0.011 - 0.059) | | | |
| Mbarara | 264 | 0.104 (0.068 - 0.141) | | 0.087 (0.062 - 0.112) | | | | | 0.102 (0.086 - 0.118) | | | |
| Ntungamo | 225 | 0.145 (0.000 - 1.000) | | 0.079 (0.049 - 0.108) | | | | | 0.089 (0.055 - 0.122) | | | |
| Rubirizi | 57 | 0.074 (0.037 - 0.112) | | 0.074 (0.049 - 0.099) | | | | | 0.065 (0.040 - 0.090) | | | |
| Rukungiri | 181 | 0.092 (0.051 - 0.133) | | 0.082 (0.056 - 0.107) | | | | | 0.052 (0.006 - 0.098) | | | |
| Sheema | 180 | 0.031 (0.000 - 0.713) | | 0.053 (0.024 - 0.081) | | | | | 0.022 (0.000 - 0.051) | | | |
| **West Nile** |  |  |  | |  |  |  |  |  |  |  |  |
| Arua | 1410 | 0.035 (0.025 - 0.044) | | 0.034 (0.024 - 0.044) | | | | | 0.042 (0.038 -0.047) | | | |
| Nebbi | 587 | 0.022 (0.001 - 0.044) | | 0.035 (0.017 - 0.052) | | | | | 0.031(0.006 - 0.056) | | | |
| **Western** |  |  |  | |  |  |  |  |  |  |  |  |
| Hoima | 283 | 0.148 (0.107 - 0.190) | | 0.089 (0.063 - 0.114) | | | | | 0.137 (0.122 - 0.153) | | | |
| Kabarole | 270 | 0.022 (0.001 - 0.044) | | 0.035 (0.017 - 0.052) | | | | | 0.031 (0.006 - 0.056) | | | |
| Kamwenge | 163 | 0.019 (0.007 - 0.032) | | 0.023 (0.010 - 0.037) | | | | | 0.024 (0.012 - 0.036) | | | |
| Kasese | 419 | 0.079 (0.048 - 0.110) | | 0.070 (0.046 - 0.093) | | | | | 0.079 (0.062 - 0.097) | | | |
| Kyenjojo | 266 | 0.148 (0.107 - 0.190) | | 0.089 (0.063 - 0.114) | | | | | 0.137 (0.122 - 0.153) | | | |
